# Novel insights into the multifaceted and tissue-specific roles of the endocytic receptor LRP1

**DOI:** 10.1016/j.jbc.2024.107521

**Published:** 2024-06-29

**Authors:** Kazuhiro Yamamoto, Simone D. Scilabra, Simone Bonelli, Anders Jensen, Carsten Scavenius, Jan J. Enghild, Dudley K. Strickland

**Affiliations:** 1 Institute of Life Course and Medical Sciences, University of Liverpool, Liverpool, United Kingdom; 2 Proteomics Group of Ri.MED Foundation, Research Department IRCCS ISMETT, Palermo, Italy; 3 Department of Biological, Chemical and Pharmaceutical Sciences and Technologies, University of Palermo, Palermo, Italy; 4 Department of Molecular Biology and Genetics, Aarhus University, Aarhus, Denmark; 5 Center for Vascular and Inflammatory Diseases, University of Maryland School of Medicine, Baltimore, Maryland, USA

**Keywords:** endocytosis, ligands, interactome, secretome, extracellular matrix

## Abstract

Receptor-mediated endocytosis provides a mechanism for the selective uptake of specific molecules thereby controlling the composition of the extracellular environment and biological processes. The low-density lipoprotein receptor–related protein 1 (LRP1) is a widely expressed endocytic receptor that regulates cellular events by modulating the levels of numerous extracellular molecules *via* rapid endocytic removal. LRP1 also participates in signalling pathways through this modulation as well as in the interaction with membrane receptors and cytoplasmic adaptor proteins. *LRP1* SNPs are associated with several diseases and conditions such as migraines, aortic aneurysms, cardiopulmonary dysfunction, corneal clouding, and bone dysmorphology and mineral density. Studies using *Lrp1* KO mice revealed a critical, nonredundant and tissue-specific role of LRP1 in regulating various physiological events. However, exactly how LRP1 functions to regulate so many distinct and specific processes is still not fully clear. Our recent proteomics studies have identified more than 300 secreted proteins that either directly interact with LRP1 or are modulated by LRP1 in various tissues. This review will highlight the remarkable ability of this receptor to regulate secreted molecules in a tissue-specific manner and discuss potential mechanisms underpinning such specificity. Uncovering the depth of these “hidden” specific interactions modulated by LRP1 will provide novel insights into a dynamic and complex extracellular environment that is involved in diverse biological and pathological processes.

The low-density lipoprotein (LDL) receptor family consists of several related scavenger receptors that not only function as important cargo transporters but also inform the cell of changes in its environment by mediating signaling responses. The seven core members of the LDL receptor family are structurally related and include the LDL receptor (1), very low–density lipoprotein receptor (2), low-density lipoprotein receptor–related protein 1 (LRP1) (3), LRP1b (4), LRP2/megalin (5), LRP4/MEGF7 (6), and LRP8/apoE receptor-2 (7, 8). All family members are a type I transmembrane protein and contain a short cytoplasmic tail between 50 and ∼200 amino acids. Distantly related genes encoding cell surface proteins that share some but not all of the structural elements that distinguish the core members of the LDL family include the Wnt receptor LRP6 (9), the closely related LRP5 (10, 11), and SORL1 (LR11/SorLA) (12, 13, 14, 15, 16) for review).

LRP1 was originally identified as an endocytic receptor for apoE (3) and complexes of proteases with α_2_-macroglobulin (α_2_M) (17, 18, 19). It is synthesized as a 600-kDa protein, followed by proteolytic processing by furin in the Golgi apparatus (20). This cleavage results in two, noncovalently bound polypeptide subunits—a 515-kDa heavy chain containing the extracellular ligand–binding domains (α subunit) and an 85-kDa light chain containing a transmembrane domain and a short cytoplasmic tail (β subunit) (Fig. 1). The LRP1 α subunit contains cysteine-rich complement-type repeats, commonly referred to as ligand-binding repeats, which occur in four clusters (termed I–IV). Interactions between LRP1 and many of its known ligands have been mapped to these clusters. The clusters are separated by epidermal growth factor precursor homology domains and six YWTD repeats, which forms β-propeller structures (21). The β subunit contains a cytoplasmic domain with two NPXY motifs, one YXXL motif, and two dileucine motifs (3). The YXXL motif, but not the two NPXY motifs, plays a major role in targeting LRP1 to clathrin-coated vesicles (22). The distal dileucine motif also contributes to its endocytosis, and its function is independent of the YXXL motif. On the other hand, the NPXY motifs serve as docking sites for cytoplasmic adaptor proteins (23, 24, 25).

**Figure 1:**
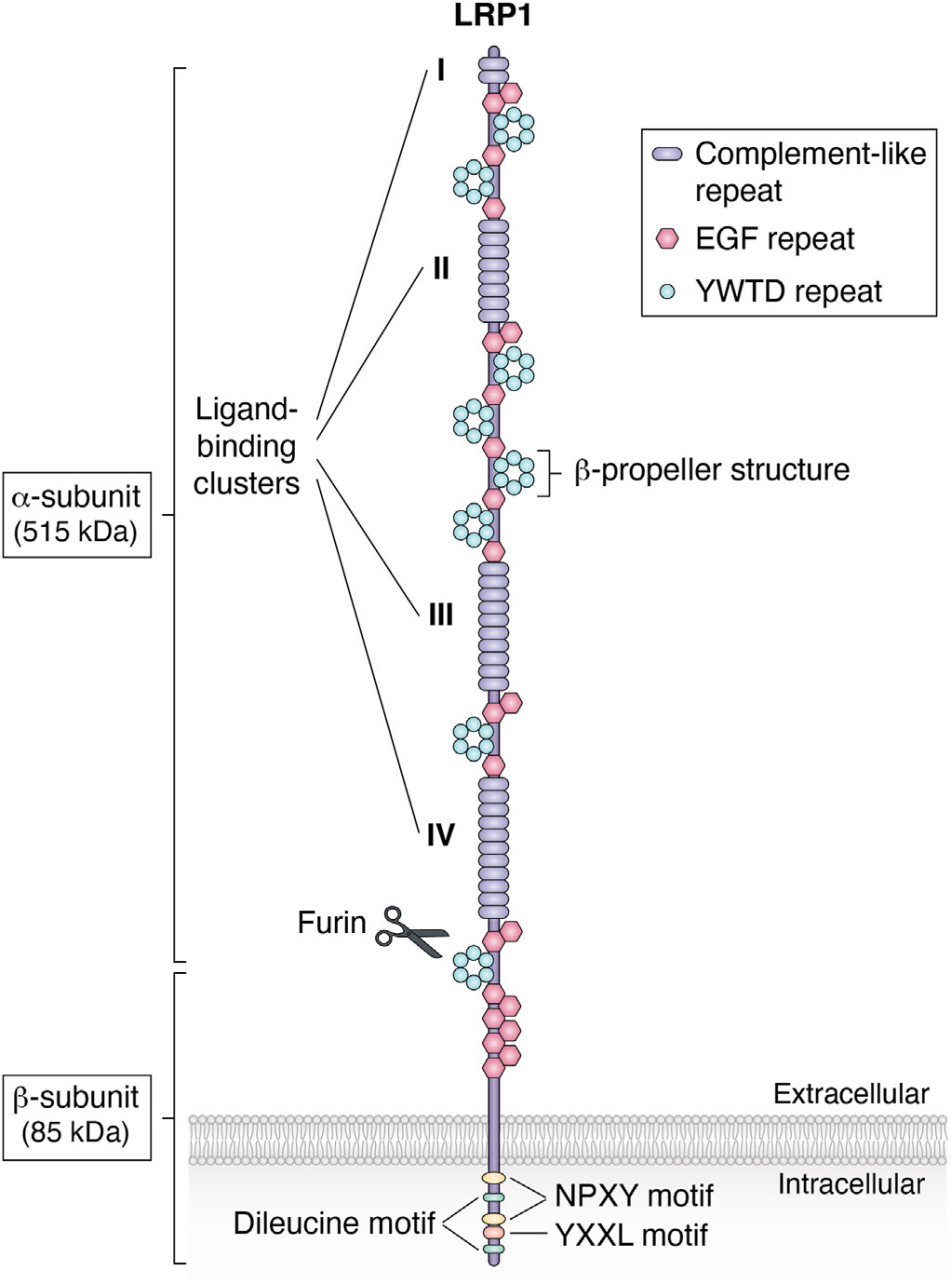
**The domain structure of LRP1.** LRP1 is synthesized as a 600-kDa protein and cleaved by furin in the Golgi apparatus resulting in two, noncovalently bound α subunit (515-kDa) and β subunit (85-kDa). The α subunit contains cysteine-rich complement-type repeats, commonly referred to as ligand-binding repeats, which occur in four clusters (termed I–IV). Interactions between LRP1 and many of its known ligands have been mapped to these clusters. The clusters are separated by epidermal growth factor (EGF) precursor homology domains and six YWTD repeats, which forms a β-propeller structure. The β subunit contains two NPXY motifs, one YXXL motif and two dileucine motifs. The NPXY motifs serve as docking sites for cytoplasmic adaptor proteins, whereas the YXXL motif plays a leading role in targeting LRP1 to clathrin-coated vesicles. The distal dileucine motif also contributes to its endocytosis independently of the YXXL motif. LRP1, low-density lipoprotein receptor–related protein 1.

LRP1 is widely expressed in different tissues and cell types (26, 27) with particularly abundant expression in hepatocytes, adipocytes, neurons, vascular smooth muscle cells, fibroblasts, macrophages, chondrocytes (28), and skeletal progenitor cells (29). Global deletion of the *Lrp1* gene in mice results in early embryonic lethality at embryonic stage E13.5 (30, 31), resulting from loss of recruitment and maintenance of mural cells to the vasculature (11). Heterozygous global *Lrp1* KO mice display cardiovascular alterations and heart weight changes (32). Tissue-specific *Lrp1* deletion in mice has revealed various biological roles of LRP1 in lipoprotein metabolism (33, 34), insulin signalling (35), inflammation (36, 37), bone development and remodelling (32, 38, 39, 40), heart development (41), and vascular wall integrity and remodeling (42, 43, 44, 45). Recent studies further revealed a critical role of skeletal progenitor LRP1 in limb development (29). Together, these studies highlight a critical, nonredundant and tissue-specific role of LRP1 in development as well as adult tissue homeostasis. However, exactly how LRP1 regulates so many distinct and specific processes is not fully clear at present. In humans, a novel autosomal recessive LRP1-related syndrome has been identified which features cardiopulmonary dysfunction, bone dysmorphology, and corneal clouding (32). The two identified siblings had compound heterozygous missense mutations, (p.Cys3807Ser and p.Val4136Gly) in LRP1. Recent work on LRP1 corneal protein interaction networks provided additional evidence of its involvement in corneal transparency and structure, aligning with these observations (46). This research exemplifies the power of studying protein interactomes, like that of LRP1, in revealing complex molecular mechanisms underlying conditions like corneal clouding, thereby opening avenues for targeted therapeutic strategies.

The objective of this review is to highlight the remarkable ability of this receptor to regulate secreted molecules in a tissue-specific manner, thereby giving insight into the complexities of physiological processes and disease mechanisms. We will reanalyze and compare the most recent mass spectrometry–based proteomics reports that systematically identified a number of molecules that interact with LRP1 (LRP1 interactome) or are either regulated or modulated by LRP1 (LRP1-controlled secretome) in various tissues. This review will also discuss methodologies and their challenges for LRP1 interaction studies and possible mechanisms underpinning the cell type and tissue specificity.

## A major role for LRP1 in endocytosis and cellular signalling

Cells constantly internalise large numbers of molecules from the cell surface and microenvironment, storing them inside the cell, recycling them back to the extracellular milieu, or degrading them in lysosomes. In eukaryotic cells, most internalized transmembrane protein receptors and ligands enter by clathrin-mediated endocytosis. Clathrin-coated vesicles assemble at the plasma membrane by forming clathrin-coated pits that mature into fully formed coated vesicles and undergo scission *via* the large GTPase, dynamin (47). LRP1 mediates clathrin-dependent endocytosis of a structurally and functionally diverse array of molecules including lipoproteins, extracellular matrix (ECM) proteins, growth factors, proteinases, proteinase inhibitors, and secreted intracellular proteins (15, 16). Previous studies demonstrated that LRP1 plays an important role in the turnover of ECM components in articular cartilage by mediating endocytic clearance of cartilage-degrading proteinases including a disintegrin and metalloproteinase with thrombospondin motifs (ADAMTS)4 (48), ADAMTS5 (28) and matrix metalloproteinase (MMP)13 (49) and their endogenous inhibitor the metalloproteinases and tissue inhibitor of metalloproteinase (TIMP)3 (50, 51). Aggrecanase-mediated aggrecan degradation in normal cartilage explants was enhanced when LRP1-mediated endocytosis was blocked (49), suggesting that LRP1 is a critical regulator of proteolytic activity of aggrecanases. Furthermore, published data thus far reveal that complexes of proteases and their target inhibitors bind tighter to LRP1 than either component alone. For example, LRP1 directly interacts with plasminogen activator inhibitor-1 (PAI-1), a SERPIN that regulates the activity of two plasminogen activators, urokinase-type plasminogen activator (uPA), and tissue-type plasminogen activator. The binding affinities of PAI-1 and uPA alone for LRP1 are approximately 100-fold weaker than that of the uPA and PAI-1 complex (30, 52, 53, 54). We also found that the binding affinities of the MMP1 and TIMP1 complex for LRP1 are approximately 30-fold higher than that of either component alone (55). The pro-MMP2/TIMP2 complex has a higher affinity for LRP-1 than either pro-MMP2 or TIMP2 alone (56). TIMP3 also facilitates the binding of MMP13, ADAMTS4, the ectodomains of MMP14, ADAM10, and ADAM17 to LRP1 (57). In addition, native forms of α_2_M are not recognized by LRP1, whereas the α_2_M/protease complex binds to LRP1 with nanomolar affinity (18). These studies suggest that LRP1 also acts as an effective mechanism for clearance of inactivated proteinase and inhibitor complexes.

Most secreted LRP1 ligands are degraded intracellularly following internalisation, and thus, many of them are rarely detectable in the tissue unless endocytosis mediated by LRP1 is blocked. These ligands likely function for a very short, finite period to maintain normal homeostatic balance in the tissues. Some ligands, however, are recycled back to the extracellular milieu, facilitating their distribution and availability (29, 58, 59). LRP1 also has the ability to interact with various cell surface receptors as well as scaffolding and adaptor proteins including Fe65 (60, 61), PSD-95 (62), Rab3a, Napg, ubiquitin b, and NYGGF4 (63). The diverse interactions and trafficking properties of LRP1 allude to its important regulatory functions in modulating the availability of biologically active secreted molecules, thus impacting their cellular signaling pathways. Additionally, the LRP1 intracellular domain contains two tyrosine phosphorylation sites (NPXY motifs). These motifs provide binding sites for a set of signaling proteins (25, 64, 65, 66). Knock-in mouse models were used to study the role of the NPXY motifs, which were independently mutated to prevent phosphorylation. The results showed that inactivation of the proximal but not distal NPXY motif results in a late fetal destruction of liver causing perinatal death (67), indicating the essential role of phosphorylation of the proximal NPXY motif in liver development.

## LRP1 dysregulation in pathological conditions

Advances in genome-wide association studies have revealed that *LRP1* SNPs are associated with several diseases, including coronary heart disease (68), abdominal aortic aneurysm (69, 70), and migraines (71, 72). *LRP1* SNPs have also been associated with a decrease in bone mineral density and content (73), carbohydrate metabolism (74), and pulmonary functions (75). Many of the *LRP1* SNPs are located within introns, and the effect of these polymorphisms on LRP1 expression and function is currently unknown. Recently, a genomic mutational constraint map using variation over 76,000 human genomes reveal that loss-of-function mutations in *LRP1* are highly significantly underrepresented (76), suggesting strong selection against haploinsufficiency for *LRP1*. A recent study by Yan *et al.* (77) identified mutations in *LRP1* in developmental dysplasia of the hip patients and these missense mutations results in reduction of LRP1 protein levels thereby inducing loss of LRP1 function. In contrast, LRP1 expression increases during hypoxia, ischemia, and tissue injury ((78) for review). Preclinical studies using various mouse models have further provided evidence for the role of LRP1 in atherosclerosis (36, 37, 45, 79, 80), aneurysm formation (43, 45, 81), undernutrition (82), osteoporosis (38, 39), developmental dysplasia of the hip (77), and Alzheimer's disease (83, 84, 85). Our recent study showed that inhibition of LRP1-mediated endocytosis in human chondrocytes results in cell death, alteration of the entire secretome, and transcriptional modulation, further highlighting the extent of LRP1 interactions and impact of its dysregulation (86).

Numerous membrane-anchored proteins are released from the cell surface by the process of regulated proteolysis called ectodomain shedding, and the enzymes responsible for shedding are primarily membrane-anchored proteinases (87, 88). Posttranslational regulation of LRP1 by proteolytic shedding regulates a wide variety of cellular and physiologic functions, and dysregulated shedding is linked to various diseases including rheumatoid arthritis, systemic lupus erythematosus (89), neuro-inflammation (90), acute respiratory distress syndrome (91), obstructive sleep apnoea (92) and cancer (93, 94, 95, 96) for reviews). LRP1 shedding triggers γ-secretase–dependent release of the LRP1 intracellular domain from the plasma membrane and its translocation to the nucleus, where it modulates the function of interferon regulatory factor 3 (61, 97). However, the exact pathological role of LRP1 shedding in these diseases is incompletely understood. Previous studies showed that proteolytic ectodomain shedding of LRP1 is increased in articular cartilage obtained from patients with osteoarthritis (OA). This process is shown to be mediated by the membrane-bound metalloproteinases, MMP14 and ADAM17 (28, 98). LRP1 shedding effectively impairs the endocytic capacity of the cell by reducing the levels of cell-surface LRP1 and by converting membrane-anchored LRP1 into soluble decoy receptors, resulting in the prolonged presence of LRP1 ligands in the tissue. We also showed that soluble shed LRP1 ectodomain competitively inhibits endocytosis of ADAMTS5 and enhances its aggrecanase activity (98). Furthermore, the soluble shed LRP1 ectodomain can diffuse and may affect distant cells and tissues. These studies reveal a difference between LRP1 deficiency and an increase in LRP1 shedding, urging careful interpretation of the studies that manipulate LRP1 levels.

## Therapeutic potential to recover LRP1 function and target specific LRP1 interactions

Inhibition of ADAM17 and MMP14 blocks LRP1 shedding, rescues the endocytic capacity of the cell and reduces ECM degradation in OA cartilage (98), indicating that OA chondrocytes can recover LRP1 function. Furthermore, chondrocyte-specific deletion of Adam17 reduced cartilage degradation in mouse OA models (99). Both MMP14 and ADAM17 are biologically important in the release of growth factors and cell surface receptors in many cell types (87, 100). A recent study by Xia *et al.* showed that global, but not chondrocyte specific, MMP14 deficiency in adult mice causes inflammatory arthritis (101). Systematic inhibition of MMP14 and ADAM17 would therefore likely result in side effects. Given that protein levels of ADAM17 and MMP14 were not significantly changed between healthy and OA cartilage (98), it is likely that LRP1 shedding by these enzymes is triggered posttranslationally in OA cartilage. Inhibiting the activation of MMP14 and ADAM17, or finding agents capable of selectively interfering with their interaction with LRP1 while preserving their physiological functions, presents an attractive approach to slowing down cartilage destruction in the development of OA. However, more studies are required to explore this potential.

LRP1 modulates inflammatory responses ((102, 103, 104) for review). In macrophages, NF-κB activation induces expression of complement proteases, plasminogen activators, and inflammatory mediators. LRP1 and ligand interaction reduces the cell-surface levels of tumour necrosis factor receptor-1 and attenuates activation of IκB kinase and NF-κB signaling in macrophages (105). Toldo *et al.* developed a synthetic LRP1-binding oligopeptide (FVFLM), termed SP16, which acts as LRP1 agonist (106). SP16 is derived from a region of α1-antitrypsin and inhibits NF-κB signaling induced by lipopolysaccharide (LPS) or Gp96 in THP-1 human monocytic cell line. Furthermore, SP16 showed a powerful anti-inflammatory cardioprotective effect in acute myocardial infarction mouse models (106). This oligopeptide also showed efficacy in rodent models of acute and neuropathic pain (107), emphasising its translational value. Nonpathogenic cellular prion protein (PrPC) was previously reported to demonstrate anti-inflammatory activity in a variety of contexts, including in experimental autoimmune encephalitis and in ischemic brain injury ((108) for review). LRP1 functions as a receptor for nonpathogenic cellular PrPC (109, 110, 111, 112). A recent study by Mantuano *et al.* designed a synthetic LRP1-binding oligopeptide named P3, which has been designed based on a LRP1 recognition motif of PrPC (113). They demonstrated that P3 interacts with LRP1, inhibits LPS-induced cytokine expression in macrophages and microglia, and rescues the increased susceptibility of cellular PrPC-deficient mice to LPS (113). Considering that inflammatory conditions increase LRP1 shedding, combination of LRP1 shedding blockade and anti-inflammatory synthetic LRP1-binding oligopeptide may be effective approach to dampen inflammation. However, application of LRP1 agonists for anti-inflammatory therapy needs to be carefully considered and requires further investigation as LRP1 has been shown to have proinflammatory effects in fibroblasts. Binding of SERPINE2 to LRP1 increases collagen deposition through activation of ERK1/2 and β-catenin signaling pathways in myocardial fibroblasts (114, 115).

## The LRP1 interactome

To date, more than 70 secreted LRP1 ligands have been reported in the literature (16, 86, 96, 116, 117) and the list is still growing (Table 1). It is likely that several secreted LRP1 ligands are rarely detectable in cell culture and tissue due to their rapid endocytic clearance. Hence, inhibition of LRP1-mediated endocytosis is crucial to identify tightly regulated LRP1 ligands. Receptor-associated protein (RAP) is a 40-kDa molecular endoplasmic reticulum–resident chaperon that binds to the ligand-binding region of LRP1 with *K*_*D*_ values within a subnanomolar range (118, 119). Endogenous RAP assists in LRP1 folding and trafficking to the cell surface (120, 121). Addition of purified RAP to cell or tissue culture is widely used to competitively block the interaction between LRP1 and its ligands but RAP also binds to other LDL receptor family members (122, 123, 124). The purified soluble form of full-length LRP1 (sFL-LRP1) or recombinant soluble LRP1 ligand-binding clusters (86, 116) binds to LRP1 ligands and competes with endogenous LRP1 for the binding to LRP1 ligands. Previous studies showed that the recombinant soluble form of LRP1 (sLRP1) containing the N-terminal half of the binding cluster II (sLRP1-II-N) preferentially binds to TIMP3 and midkine over metalloproteinases including ADAMTS5, MMP2, MMP9, and MMP13 (125). Although LRP1 ligand-binding clusters II and IV are responsible for most of the known ligand binding (48, 49, 125, 126, 127, 128), these clusters may not entirely share ligands with FL-LRP1. At present, the combination of gene silencing or genetic deletion of LRP1 and coimmunoprecipitation (co-IP) with sFL-LRP1 followed by mass spectrometry is likely to be the most specific and powerful approach to identify a diverse array of LRP1 ligands. We recently established a novel and simplified purification protocol for sFL-LRP1 based on its affinity for RAP that produces significantly higher yields of authentic LRP1. In the original LRP1 purification protocol (18), α_2_M activated by methylamine treatment was used as the affinity ligand during the column purification. Methylamines react with the α_2_M thioester bond, leading to large conformational changes that expose the LRP binding site at the C terminal of the molecule (129). This protocol (18) yields approximately 50–100 μg of LRP1 per human placenta, while the novel RAP protocol (46) results in significantly higher yields, producing 500–750 μg of LRP1 per human placenta. However, since RAP binds all LDL receptor family members, other LDL receptor family members may copurify with LRP1 using this procedure.

**Table 1:** List of representative secreted proteins whose direct interaction with LRP1 and/or

Name	Gene name	Cell type/tissue	Reference
α1-Antitrypsin	SERPINA1	Human macrophages	(185)
α_2_-Macroglobulin (either complexed with proteases or activated by methylamine)	A2M	Human/rat/mouse liver Human monocytes Human placenta Rabbit macrophages Rat/mouse hepatocytes Human hepatoma cell line (HepG2) Rat embryonal carcinoma cell line (L2p58) Rat kidney fibroblast cell line (NRK-2T) Mouse embryonic fibroblast cell line (MEF)	(17, 18, 19, 172, 186, 187, 188, 189, 190)
α-Synuclein	SNCA	Mouse brain Human induced pluripotent stem cells–derived neurons	(191)
ADAMTS1	ADAMTS1	Human articular chondrocytes MEF	(86)
ADAMTS4	ADAMTS4	Porcine articular cartilage Human articular chondrocytes MEF	(48)
ADAMTS5	ADAMTS5	Human/procine articular cartilage Human articular chondrocytes MEF	(28, 57, 192, 193)
Amyloid β peptide	APP	Human brain MEF	(194, 195)
Amyloid precursor protein	APP	MEF	(154)
ApoE/ApoE-containing lipoproteins	APOE	Human/mouse liver Rat retinal ganglion cells Human skin fibroblasts HepG2 Chinese hamster ovary cell line (CHO)	(3, 196, 197, 198, 199)
Bone morphogenetic protein-binding endothelial cell precursor–derived regulator	BMPER	Mouse endothelial cells MEF	(200)
Calreticulin	CALR	Human monocyte–derived macrophages Bovine aortic endothelial cell line (BAE) Mouse macrophage cell line (J774) MEF	(201, 202)
C4b-binding protein	C4BPA	MEF	(144)
CCN1/cysteine-rich angiogenic inducer 61 (CYR61)	CCN1	Human skin fibroblasts	(203)
CCN2/connective tissue growth factor	CCN2	Mouse articular cartilage Mouse developing limb Mouse aorta Human chondrosarcoma cell line (HCS-2/8) Human osteosarcoma cell line (MG3) Rat hepatic stellate cells Mouse adipocyte cell line (BMS2) MEF	(29, 43, 58, 86, 145, 204, 205)
Cell migration–inducing and hyaluronan-binding protein/KIAA1199	CEMIP	Mouse articular cartilage Human articular chondrocytes MEF	(86)
Chylomicron remnants	APOB: APOE: APOA1	Mouse plasma Mouse liver	(198, 206)
*Clostridium perfringens* TpeL toxin	tpeL	Human epithelial cell line (Hela) MEF African green monkey kidney epithelial cell line (Vero)	(207)
Clusterin	CLU	Human breast cancer cell line (MCF-7) Human metastatic breast cancer cell line (MDA-MB-231)	(208)
Coagulation factor VIII	F8	Mouse plasma CHO MEF	(209, 210)
Coagulation factor Xa: tissue factor pathway inhibitor (TFPI) complexes	F10: TFPI	HepG2 MEF	(211)
Coagulation factor XIa: nexin-1 complexes	F11: SERPINE2	Human foreskin fibroblasts Mouse cerebellar granular neuron precursors MEF	(212, 213, 214)
Complement component 3	C3	Human fibroblasts MEF	(215)
Decorin	DCN	Mouse skeletal muscle cell line (C2C12) CHO	(216, 217)
Fibronectin	FN1	CHO MEF	(218)
Glia-derived nexin (nexin1)	SERPINE2	Mouse cerebellar granular neuron precursors MEF	(151, 214)
Heat shock protein 70	HSPA1A	Mouse macrophage-like cell line (RAW264.7)	(219)
Heat shock protein 90-α	HSP90AA1	Human dermal fibroblasts MCF-7 MDA-MB-231 RAW264.7	(208, 219, 220)
Hemopexin and its complex with heme	HPX	African green monkey kidney fibroblast-like cell line (COS-1)	(221)
Hepatic lipase	LIPC	HepG2 MEF	(222)
Hepatocyte growth factor activator	HGFAC	Only direct interaction validated *in vitro*	(86)
High mobility group protein B1	HMGB1	Only direct interaction validated *in vitro*	(86)
High mobility group protein B2	HMGB2	Human articular chondrocytes MEF	(86)
HIV-Tat protein	tat	Human neurons Rat pheochromocytoma cell line (PC12)	(223)
Serine protease HtrA1	HTRA1	Mouse aorta MEF	(43)
Insulin-like growth factor–binding protein 3	IGFBP3	Mink lung epithelial cell line (Mv1Lu) MEF	(224)
Insulin-like growth factor–binding protein 7	IGFBP7	Human articular chondrocytes MEF	(86)
Lactoferrin	LTF	Human skin fibroblasts	(225, 226)
Leptin	LEP	Mouse hypothalamic GnRH neuronal cell line (GT1-7)	(227)
Lipoprotein lipase	LPL	Human skin fibroblasts HepG2	(228, 229)
Macrophage migration inhibitory factor	MIF	HEK293	(134)
Matrix metalloproteinase (MMP)1	MMP1	Human aortic smooth muscle cells Human chondrosarcoma cell line (HTB94)	(55, 57)
MMP2	MMP2	Mouse skin fibroblasts Human fibrosarcoma cell line (HT1080)	(56, 230)
MMP9	MMP9	MEF	(55, 231)
MMP13	MMP13	Human articular chondrocytes Rat osteosarcoma cell line (UMR 106-01) MEF	(57, 86, 232)
Midkine	MDK	Mouse cortex neurons	(233)
Neuroserpin	SERPINI1	Mouse cortical cells MEF	(234)
Plasminogen activator inhibitor (PAI-1)	SERPINE1	MEF	(53)
Pregnancy zone protein: protease (chymotrypsin, trypsin, or tPA) complex	PZP	Only direct interaction validated *in vitro*	(235, 236)
Procathepsin D	CTSD	Human mammary fibroblasts MEF	(86, 237)
Prosaposin	PSAP	Human fibroblasts Mouse plasma Mouse fibroblasts Rat hepatoma cell line (FTO2B) PC12 Mouse sertoli cell line (TM4)	(238)
*Pseudomonas* exotoxin A	eta	Mouse fibroblast cell line (L-M) MEF	(239, 240)
Slit homolog 2 protein	SLIT2	Mouse articular cartilage Human articular chondrocytes MEF	(86)
SPARC/osteonectin	SPARC	HEK293	(86, 134)
Tau protein	MAPT	Human neuroblastoma cell line (SH-SY5Y) CHO MEF	(198)
Thrombospondin 1	THBS1	Human saphenous vein smooth muscle cells Human lung fibroblast cell line (WI38) Human umbilical vein endothelial cells (HUVEC)	(142, 241)
Thrombospondin 2	THBS2	Mouse skin fibroblasts MEF	(230, 242)
Thrombospondin 4	THBS4	Only direct interaction validated *in vitro*	(243)
Tissue factor pathway inhibitor	TFPI	Rat hepatoma cell line (MH_1_C_1_) HepG2	(244)
Tissue inhibitors of metalloproteases (TIMP)1	TIMP1	Human aortic smooth muscle cells Human embryonic kidney cell line (HEK293) CHO Mouse cortical neurons	(55, 57, 245) (57, 134)
TIMP2	TIMP2	HT1080 HEK293	(55, 56, 57, 134)
TIMP3	TIMP3	Mouse developing limb Porcine articular chondrocytes Human glioblastoma cell line (U251) Human prostate adenocarcinoma cell line (PC3) HEK293 HTB94 COS-1 MEF	(50, 51, 55) (29, 57, 125, 134)
TIMP4	TIMP4	Only direct interaction validated *in vitro*	(55)
Tissue-type plasminogen activator (tPA)	PLAT	Only direct interaction validated *in vitro*	(246)
Transforming growth factor-β1 (TGF-β1)	TGFB1	Mv1Lu MEF	(224)
TGF-β2	TGFB2	Mouse macrophages	(80)
Triglyceride-rich lipoproteins		Rat liver Mouse plasma Human fibroblasts	(247, 248, 249)
Tumor necrosis factor–inducible gene 6 protein	TNFAIP6	Human articular chondrocytes MEF	(86)
Urokinase-type plasminogen activator (uPA)	PLAU	HepG2	(246, 250, 251)
Von Willebrand factor	VWF	Mouse plasma	(252)
Wnt5a	WNT5A	Mouse developing limb Human articular chondrocytes MEF	(29)
Wnt11	WNT11	Only direct interaction validated *in vitro*	(29)
Complexes			
α1-antitrypsin: neutrophil elastase	SERPINA1: ELANE	MEF	(172)
α1-antitrypsin: trypsin	SERPINA1: PRSS1	HepG2 MEF	(253)
ADAMTS4: TIMP3	ADAMTS4: TIMP3	HTB94	(57)
ADAMTS5: TIMP3	ADAMTS5: TIMP3	HTB94	(57)
MMP1: TIMP1	MMP1: TIMP1	Only direct interaction validated *in vitro*	(55)
MMP1: TIMP3	MMP1: TIMP3	HTB94	(57)
MMP2: thrombospondin 2 complex	MMP1: THBS2	Mouse skin fibroblasts	(230)
ProMMP9: TIMP1	MMP9: TIMP1	MEF	(55)
Proteinase-complexed C1 inhibitor	SERPING1	Mouse plasma MEF	(254)
Thrombin: antithrombin III	F2: SERPINC1	Rat plasma HepG2 COS-1 MEF	(251, 253)
Thrombin: heparin cofactor II	F2: SERPIND1	HepG2 MEF	(253)
Thrombin: nexin1	F2: SERPINE2	Human foreskin fibroblasts COS-1 MEF	(251, 255)
Thrombin: PAI-1	F2: SERPINE1	Rat pretype II pneumocytes	(256)
Thrombin: protein C inhibitor	F2: SERPINA5	COS-1	(251)
tPA: neuroserpin	PLAT: SERPINI1	Mouse cortical cells MEF	(234)
tPA: PAI-1	PLAT: SERPINE1	COS-1	(246, 251)
uPA: antithrombin III	PLAU: SERPINC1	COS-1	(251)
uPA: C1 inhibitor	PLAU: SERPING1	COS-1	(251)
uPA: nexin1	PLAU: SERPINE2	Human foreskin fibroblasts Human monocyte-like cell line (U937) Mouse fibroblast cell line (LB6)	(251, 257, 258)
uPA: PAI-1	PLAU: SERPINE1	Human monocytes PC3 COS-1	(30, 246, 251, 259)
uPA: PAI-2	PLAU: SERPINB2	PC3	(259)
uPA: protein C inhibitor	PLAU: SERPINA5	COS-1	(251)

LRP1-dependent regulation has been validated by targeted approach.

ADAMTS, a disintegrin and metalloproteinase with thrombospondin motifs; LRP1, low-density lipoprotein receptor–related protein 1; TNF, tumor necrosis factor.

### Different LRP1 extracellular interactomes in chondrocytes, cornea tissue, and Chinese hamster ovary cells

**Figure 2:**
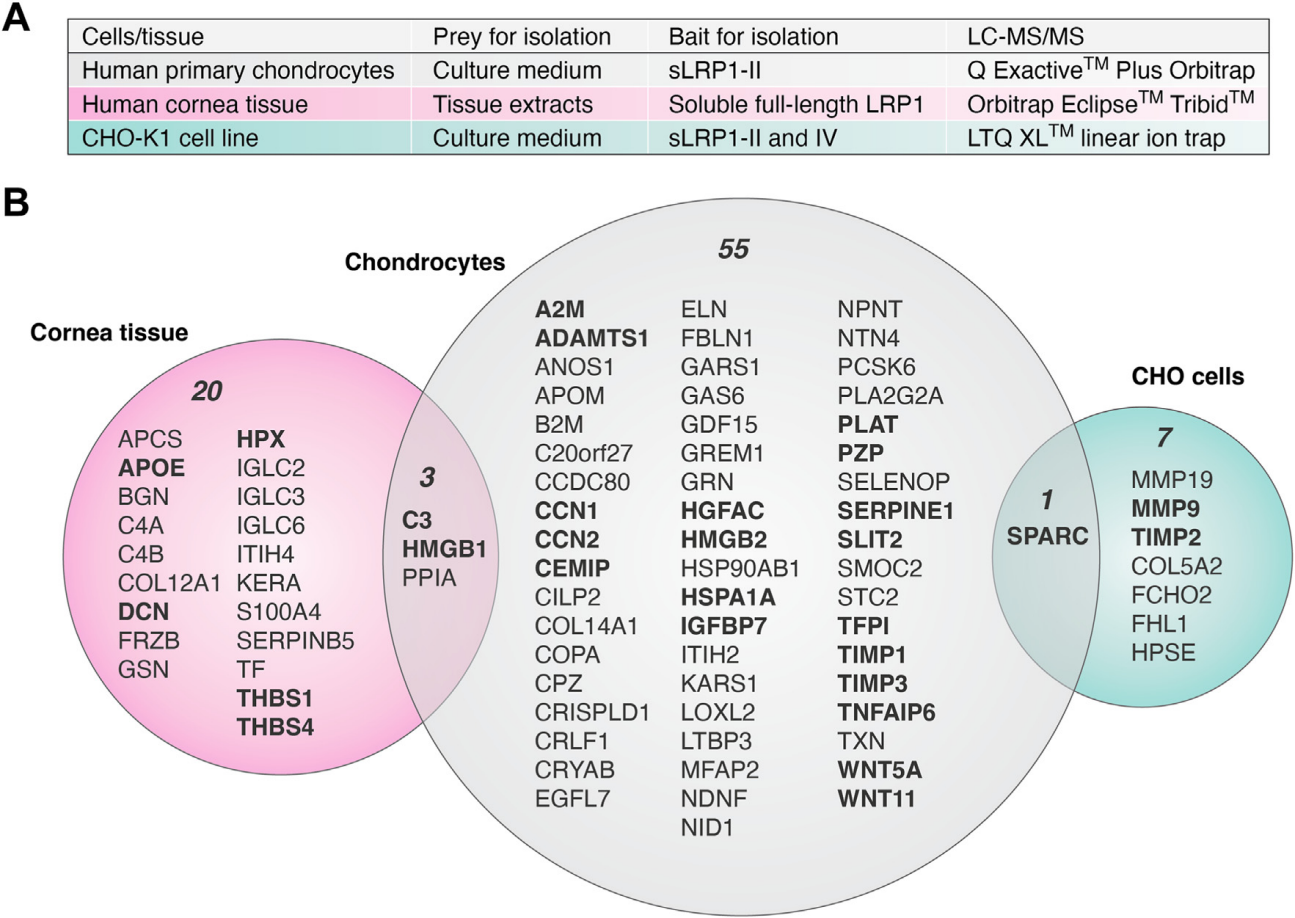
**Different LRP1 extracellular interactomes in chondrocytes, cornea tissue, and CHO-K1 cells.***A*, table showing experimental conditions for identification of the LRP1 extracellular interactome in human chondrocytes, human cornea tissue, and CHO-K1 cells. *B*, *Venn diagram* showing the total number and gene names of secreted proteins co-IP with sLRP1-II in the medium of chondrocytes, sFL-LRP1 in cornea tissue extracts, and sLRP1-II and sLRP1-IV in CHO-K1 cells. The *bold gene name* indicates the molecule whose direct interaction with LRP1 and/or LRP1-dependent regulation has been validated by targeted approach. CHO, Chinese hamster ovary; Co-IP, coimmunoprecipitation; LRP1, low-density lipoprotein receptor–related protein 1; sFL-LRP1, soluble form of full-length LRP1; sLRP1, soluble form of LRP1; sLRP1-II, sLRP1 containing binding cluster II.

In this review, we compared the three co-IP LRP1 interactome datasets (Fig. 2*A*) included in recent studies using either purified recombinant sLRP1 containing the binding cluster II (sLRP1-II) in human chondrocytes (86), purified sFL-LRP1 in human cornea tissue (46) or previous work done by Fernandez-Castaneda *et al.* using immunoglobulin Fc fusion sLRP1-II and sLRP1-IV in mouse myelin and Chinese hamster ovary (CHO)-K1 cells (116). According to UniProt annotations, a total of 59, 23, and 8 secreted co-IP proteins were identified in chondrocytes, cornea tissue, and CHO cells, respectively (Fig. 2*B*), whereas no secreted proteins were identified in myelin. Among these molecules, a total of 22, 7, and 3 molecules in chondrocytes, cornea tissue, and CHO cells, respectively, were validated for either/both direct interaction with LRP1 or/and LRP1-dependent regulation. Co-IP, solid-phase binding assay, and surface plasmon resonance have been the main assays used for the former validation. Gene-deletion/silencing of LRP1, RAP, and LRP1 neutralizing antibody have been widely used for the latter validation. A comparison of these LRP1 extracellular interactomes reveals that there are no common ligands in all three LRP1 interactomes (Fig. 2*B*). The three ligands high mobility group protein B1, peptidyl-prolyl *cis-trans* isomerase A, and complement C3 were identified in both human chondrocytes and cornea tissue. SPARC (also known as basement-membrane protein 40 and osteonectin) is the only secreted protein commonly identified as a LRP1 ligand in chondrocytes and CHO cells. No common ligands have been found between cornea tissue and CHO cells. These studies have the following limitations but support the notion that the LRP1 interactome is cell- and tissue-specific. First, the difference in the experimental methodology applied to isolate LRP1 ligands (Fig. 2*A*) may contribute to the differences among the LRP1 interactomes. Second, due to the technical challenges associated, it is likely that these studies have not provided complete list of LRP1 ligands and/or gave false positives.

**Figure 3:**
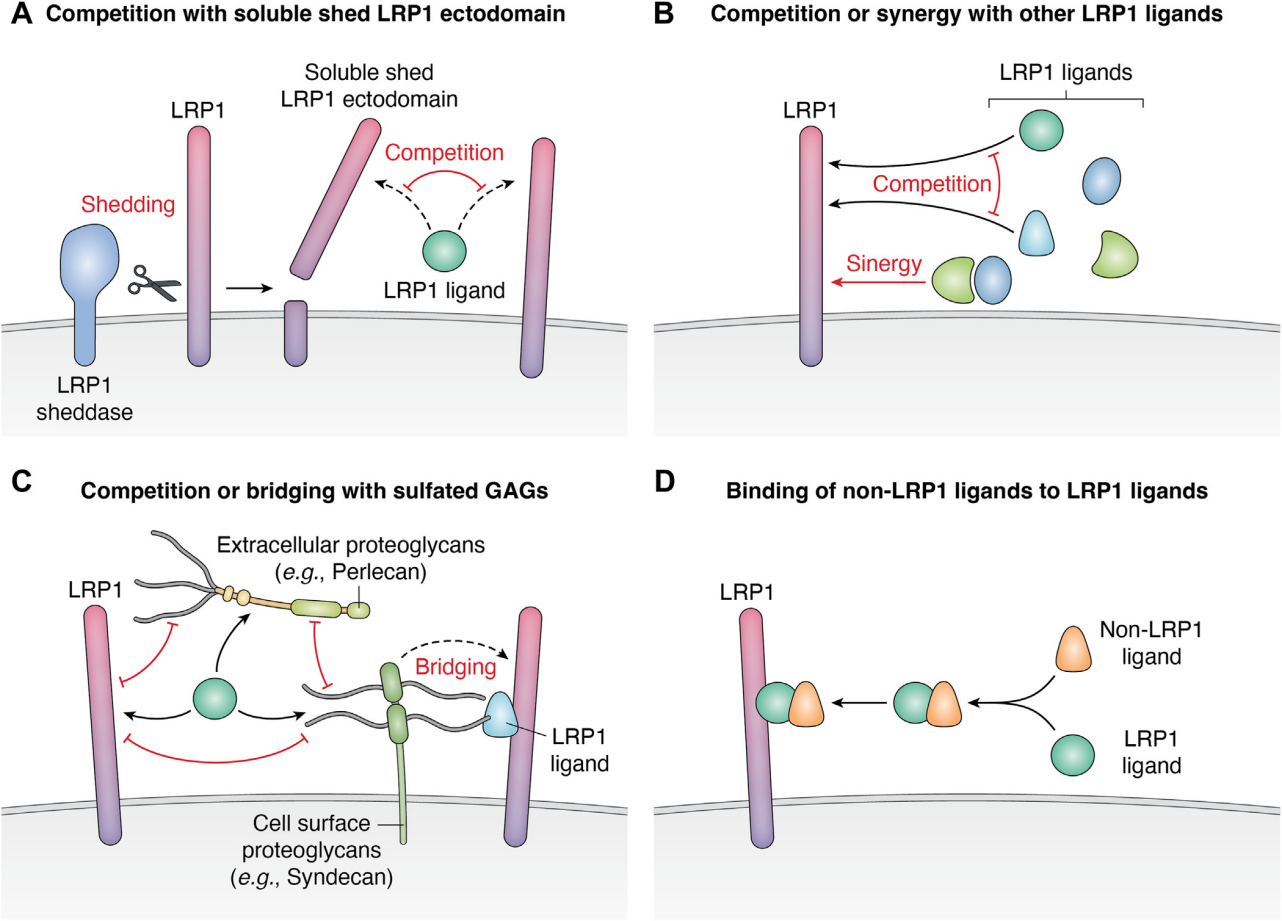
**The LRP1 interactome is modulated by a variety of mechanisms.** The LRP1 interactome can be shaped by cell-specific expression profiles and levels of secreted proteins as well as (*A*) competition with soluble shed LRP1 ectodomain, (*B*) competition or synergy with other LRP1 ligands, (*C*) competition or bridging with sulfated GAGs, and (*D*) binding of non-LRP1 ligands to LRP1 ligands. GAG, glycosaminoglycan; LRP1, low-density lipoprotein receptor–related protein 1.

The resolution of mass spectrometry instruments refers to their ability to distinguish different molecular species based on their mass-to-charge ratio. High-resolution instruments can separate closely related peptides, which is crucial for accurate protein identification. Combined with increased sensitivity of modern mass spectrometers, a deeper analytical approach will identify a larger number of potential ligands, increasing the complexity of the interactome. However, this can also escalate the chances of identifying false positive proteins that are identified as LRP1 ligands but are not functionally relevant or are artifacts of the experimental setup. High analytical depth is desirable for a comprehensive interactome, but it must be paired with proper controls in the experimental design. For instance, blank beads (without sLRP1-II) were used as a control in human chondrocytes (86), whereas Fc fragment alone and RAP were used in myelin and CHO cells (116). In cornea (46), controls included blank beads, RAP, and without Ca^2+^, which requires for LRP1 to bind to ligands. Comparing the results from these controls with the experimental conditions helps to distinguish specific interactions from nonspecific ones.

### The LRP1 interactome is modulated by a variety of mechanisms

The cell and tissue specificity of the LRP1 interactome can be modulated by several factors that include LRP1 shedding, its presence in circulation and extracellular vesicles, the concentrations and distribution of LRP1 ligands, their affinity and binding sites for LRP1, and even presence of other molecules that do not directly interact with LRP1. A variety of mechanisms that can regulate the LRP1 interactome are discussed below.

#### Competition with the soluble shed LRP1 ectodomain

As described above, the soluble shed LRP1 ectodomain binds to LRP1 ligands and competes with membrane-bound LRP1 for binding to LRP1 ligands (Fig. 3*A*). In tissue, conversion of membrane-anchored LRP1 into soluble decoy receptors along with reduction of the levels of cell-surface LRP1 results in the accumulation of LRP1 ligands. Furthermore, the soluble shed LRP1 ectodomain can diffuse and may affect the abundance or availability of LRP1 ligands in distant tissues. LRP1 can be also released into the circulation (130, 131) by proteolytic shedding mediated by BACE1 protease and a hepatic metalloproteinase (132). In the plasma, the concentrations of soluble forms of LRP1 are in the nano-molar range. Given that LRP1 interacts with a variety of distinct molecules in the circulation including proteinase–inhibitor complexes, activated coagulation factors and chylomicron remnants (16, 117, 133), most circulating LRP1 may be occupied with ligands.

#### Competition or synergy with other LRP1 ligands

Competition among LRP1 ligands likely contributes to the cell and tissue specificity of the LRP1 interactome (Fig. 3*B*). For example, previous studies demonstrated that overexpression of TIMP3, which has *K*_*D*_ values for LRP1 within the nanomolar range (125), induces extracellular accumulation of several LRP1 ligands (134). We have previously shown a correlation between the affinity of ligands for LRP1 and their rate of endocytosis (48, 86). For example, the 10-fold higher concentration of ADAMTS5 inhibits endocytosis of ADAMTS4 by chondrocytes due to the much higher affinity of ADAMTS5 for LRP1 than that of ADAMTS4 (48). Although both MMP13 and ADAMTS4/5 bind to sLRP1-II, MMP13 does not interfere with the binding of ADAMTS4/5 to sLRP1-II, even though it has high-affinity binding constants in the range of 2.7 to 6.0 nM. This suggests that MMP13 and ADAMTS4/5 bind to different sites within the cluster II.

#### Competition or bridging with sulfated glycosaminoglycans

Several LRP1 ligands bind to sulfated glycosaminoglycans (GAGs), with their extracellular availability determined by their relative affinity for each (88, 135) (Fig. 3*C*). Heparan sulfate and chondroitin sulfate E selectively regulate post-secretory trafficking of TIMP3 by inhibiting its binding to LRP1 (135). Heparin, a highly sulfated GAG, binds to ADAMTS4 (136), ADAMTS5 (137) and MMP13 (138), and blocks their binding to both LRP1 and ECM (135, 136, 137, 139). It should be noted that neither of these proteinases were identified in the conditioned medium of chondrocytes either in the absence or presence of sLRP1-II by our proteomics analysis (86). Although we cannot rule out the possibility that some of these enzymes were not identified by mass spectrometry due to technical limitations, a majority of them might be attached to the ECM or cell surface *via* sulfated GAGs when endocytosis mediated by LRP1 was inhibited. In contrast, several LRP1 ligands including very low–density lipoprotein (140), amyloid-beta peptides (141), thrombospondin 1 (142), the coagulation factor VIII (143), C4b-binding protein (144), and connective tissue growth factor (CTGF, also known as CCN1) (145) are shown to bind with low affinity to GAGs of cell surface heparan sulfate proteoglycans that in turn facilitate their binding to LRP1.

#### Binding of non-LRP1 ligands to LRP1 ligands

The chondrocyte LRP1 interactome includes fibulin-1C and progranulin, which do not directly bind to LRP1 (86) (Fig. 3*D*). It has been reported that fibulin-1C binds to ADAMTS1 (146) and CTGF (147), which directly bind to LRP1. Prosaposin, which is also one of the previously reported LRP1 ligands, interacts with progranulin and regulates its levels *in vitro* and *in vivo via* LRP1-mediated endocytosis (148, 149). It is thus likely that these non-LRP1 ligands are regulated by LRP1 *via* direct interaction with LRP1 ligands. The cornea LRP1 interactome includes biglycan (46) but LRP1 has not to our knowledge been reported to interact with biglycan. As biglycan binds to the known LRP1 ligand apoB (150), which was also identified in the cornea LRP1 interactome, biglycan may bind to LRP1 *via* apoB.

### The interaction of LRP1 with transmembrane proteins

On the cell surface, LRP1 interacts with HS proteoglycans (140) including syndecan-1 (151) and glypican-3 (152). These interactions with HS proteoglycans regulate the binding of secreted LRP1 ligands to LRP1 and *vice versa* and also regulate cellular signaling events ((16) for review). In addition, LRP1 interacts with CD44 (153) as well as amyloid precursor protein (154, 155), where LRP1 modulates the production of the amyloid-β peptide. LRP1 also interacts with several integrins including αM, αL (156), β1 (157, 158, 159), and β2 (160, 161) subunits, and αMβ1 (162), αMβ2 (156), αVβ3, and αVβ5 complexes (163). The interactions with integrins regulate activity and availability of the integrins, thereby impacting cell attachment and migration. Consequently, the interaction of LRP1 with integrins emerges as a potential candidate target for preventing cancer invasion (96). The interaction of LRP1 with secreted ligands also regulates the availability of cell-surface receptors. For example, LRP1 regulates the availability of uPA receptor (uPAR) *via* interactions with uPA and PAI-1 complexes (30, 54, 164, 165). The uPA and PAI-1 complex is a bivalent ligand (54), which triggers uPAR internalization and regulates uPAR signaling by bridging uPAR and LRP1 extracellularly. The direct interaction of LRP1 and uPA/PAI-1–occupied uPAR could mediate internalization of the occupied uPAR and subsequent recycling of unoccupied uPAR (166).

### The interaction of LRP1 with intracellular proteins

Previous work done by Fernandez-Castaneda *et al.* demonstrated that LRP1 plays a role in the clearance of cellular debris in mouse myelin (116). By employing decoy receptors sLRP1-II and IV to co-IP ligands, this study identified a total of 20 proteins by co-IP with sLRP1-II and IV from myelin extracts, all of which were intracellular proteins. Recent study identified a total of 276 proteins by co-IP with sLRP1-II from the medium of human chondrocytes out of which 50 molecules were secreted proteins (according to UniProt annotations). Most of the remaining proteins were intracellular proteins, supporting the role of LRP1 in clearing cellular debris. Gotthardt *et al.* systematically investigated the interactome for the cytoplasmic domain of LRP1 using yeast two-hybrid assays and identified 13 intracellular proteins including kinases, cytoskeletal proteins, and ion channels (167). Most of these proteins are adaptor or scaffold proteins that contain protein interaction domains such as PDZ domain and function in the regulation of mitogen-activated protein kinases, cell adhesion, vesicle trafficking, or neurotransmission. LRP1 also interacts with intracellular adaptor protein Fe65, which connects LRP1 to β-amyloid precursor protein (60, 61) and stimulates APP endocytosis and amyloid β generation (60). The interaction of LRP1 with intracellular adaptor protein, PSD-95, connects LRP1 to the N-methyl-D aspartate receptor (62), stimulating extracellular signal-regulated kinase1/2(ERK1/2) signaling (62). Kajiwara *et al.* further characterized the LRP1 cytoplasmic domain interactome and identified Rab3a, Napg, ubiquitin b, and NYGGF4 as novel cytosolic ligands (63).

## The LRP1-controlled secretome: extracellular proteins regulated or modulated by LRP1

The secretome is defined as the set of molecules and biological factors that are secreted by cells into the extracellular space. Given that dysregulation of LRP1 is associated with various diseases and conditions, the altered secretome upon LRP1 inhibition or deletion may result in a pathological condition. Defining cell- and tissue-specific LRP1-controlled secretomes is thus important to elucidate the role of LRP1 in biological processes and disease mechanisms. The recent chondrocyte secretome study revealed that the abundance of 23 previously reported LRP1 ligands were not altered by sLRP-II treatment (86). Furthermore, among a total of 52 chondrocyte secreted proteins that co-IP with sLRP1-II, only 21 of them were increased while the remaining proteins were either decreased or unchanged by sLRP1-II treatment. These studies reveal that not all LRP1 ligands increase upon LRP1 blockade and highlight a difference between the LRP1-controlled secretome and interactome and complex protein interaction networks.

### Different LRP1-controlled secretomes in chondrocytes, HEK293 cells, and the superior mesenteric artery tissue

**Figure 4:**
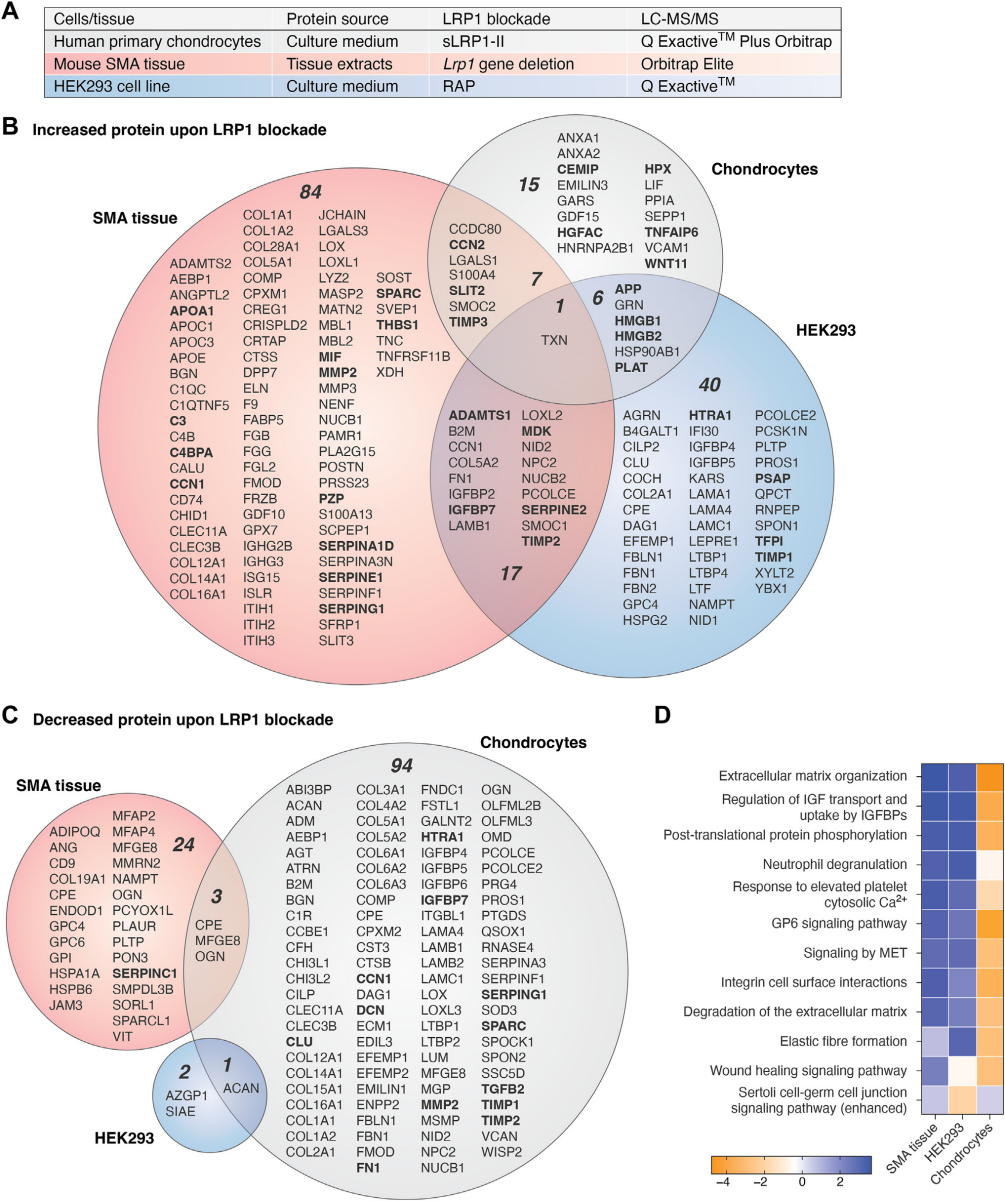
**Different LRP1-controlled secretomes in chondrocytes, HEK293 cells and the SMA tissue.***A*, table showing experimental conditions for identification of the LRP1-controlled secretome in human chondrocytes, mouse superior mesenteric artery (SMA) tissue, and HEK293 cells. *B* and *C*, *Venn diagram* showing the number of total secreted proteins and their gene names (according to UniProt annotations) that were increased (*B*) or decreased (*C*) when LRP1 and ligand interaction is blocked in chondrocytes, SMA tissue and HEK293 cells. The proteins with *p* value <0.05 and >2-fold change or only identified in either condition was counted. *Bold gene names* indicate molecule whose direct interaction with LRP1 and/or LRP1-dependent regulation has been validated by targeted approach. *D*, QIAGEN Ingenuity Pathway Analysis of the secretomes for the probable downstream effects on the canonical pathways. LRP1, low-density lipoprotein receptor–related protein 1.

We showed that inhibition of endocytosis mediated by LRP1 in human chondrocytes by sLRP1-II (86) and in HEK293 cells by RAP (134) markedly alters the secretome of these cells. Further, genetic deletion of LRP1 in vascular smooth muscle cells has a dramatic effect on secreted proteins located in superior mesenteric artery (SMA) (45). In this review, we reanalyzed and compared the three secretome datasets for chondrocytes (86), HEK293 cells (134) and SMA tissue extracts (45) (Fig. 4*A*) with threshold value >2-fold change with *p* value <0.05. Upon LRP1 blockade, a total of 29 (12), 109 (20), and 64 (13) secreted proteins were increased in the medium of chondrocytes, SMA tissue extracts and HEK293 cells, respectively, with the numbers in parentheses indicating the number of validated LRP1 ligands (Fig. 4*B*). Among them, only one protein, thioredoxin, was commonly increased in the three secretomes. Pairwise comparison showed that a total of 7, 6, and 17 proteins were commonly increased in chondrocytes and SMA secretomes, chondrocytes and HEK293 secretomes, and SMA and HEK293 secretomes, respectively. Notably, in total 15, 84, and 40 proteins were exclusively increased in chondrocytes, SMA, and HEK293 secretomes, respectively. In contrast, upon LRP1 blockade, in total 98 (12), 27 (1), and 3 (0) secreted proteins were decreased in the medium of chondrocytes, SMA tissue extracts, and HEK293 cells, respectively with the numbers in parentheses indicating the number of validated LRP1 ligands (Fig. 4*C*). A decrease in validated LRP1 ligands upon LRP1 blockade further suggests the cell-type and tissue-specific regulation of LRP1 ligands. Among them, none of the proteins were commonly decreased in three secretomes and a total of three and one proteins were commonly decreased in chondrocytes and SMA, and chondrocytes and HEK293 secretomes, respectively. Addition of purified RAP, sFL-LRP1 or sLRP1-II/IV to cell, or tissue culture inhibits LRP1-mediated endocytosis but these agents may also inhibit endocytosis mediated by other LDL receptor family members. These limitations to specifically inhibit interaction of LRP1 and its ligands, and the technical challenges associated with mass spectrometry–based proteomics may contribute to the differences among the LRP1-controlled secretomes. Nevertheless, these studies support the notion that the LRP1-controlled secretome is cell- and tissue-specific.

**Figure 5:**
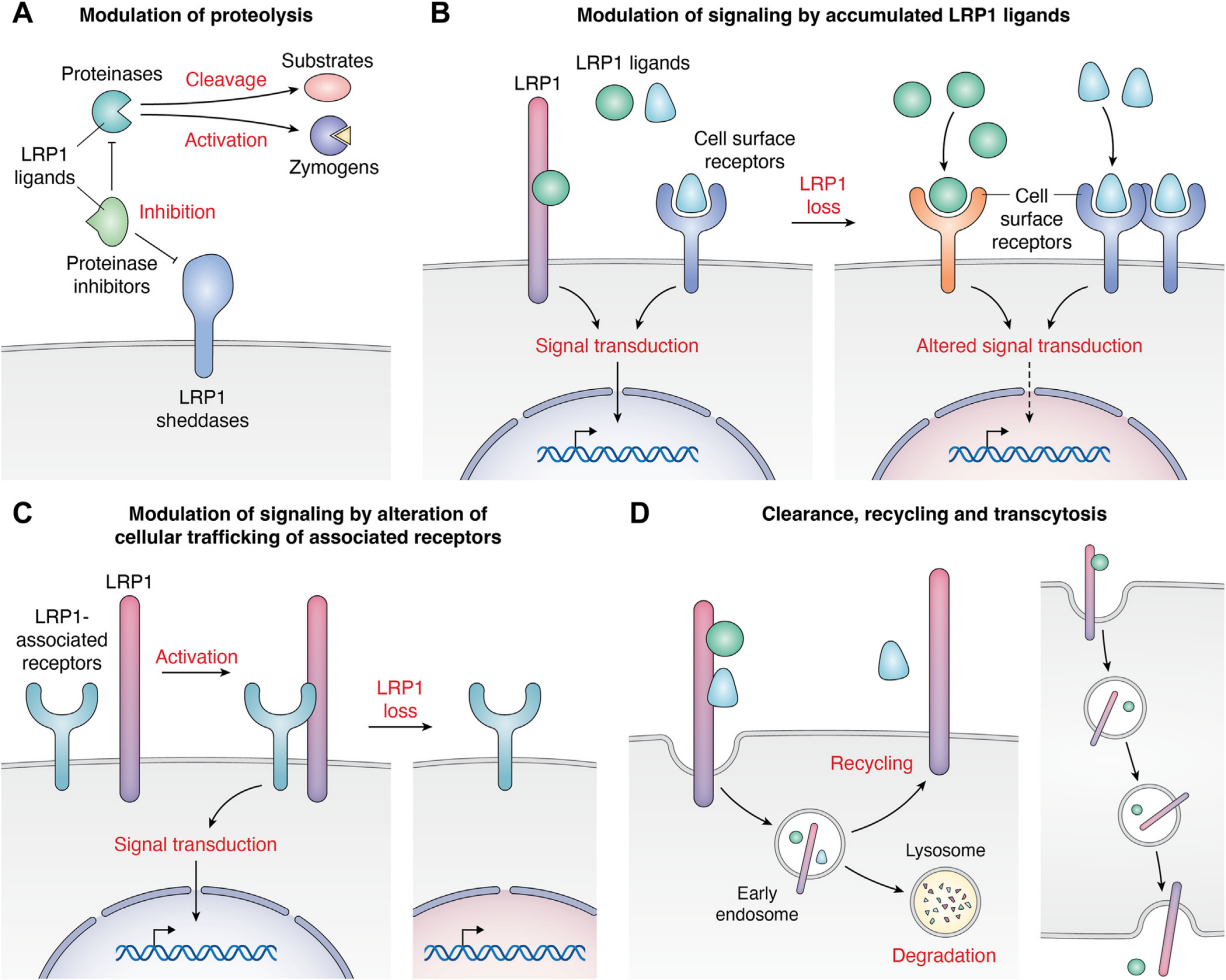
**Impact of LRP1 dysfunction on the extracellular environment and cellular signaling.** Excess activity of LRP1 ligands can modulate proteolysis of extracellular and cell membrane proteins (*A*) and cellular signaling pathways *via* the accumulation of signaling molecules (*B*). LRP1 loss also affects cellular trafficking of associated receptors, thereby alteration of cellular signaling (*C*). Endocytic recycling and transcytosis of LRP1 ligands add further complexity of the LRP1 secretome and the consequence of its dysregulation (*D*). LRP1, low-density lipoprotein receptor–related protein 1.

To elucidate the probable downstream effects of disruption of LRP1-controlled secretome, we performed causal analysis (168) of the secretomes using Ingenuity Pathway Analysis software (https://www.qiagenbioinformatics.com/products/ingenuity-pathway-analysis). Notably, the canonical pathways that can be activated upon LRP1 inhibition are remarkably similar in SMA and HEK secretomes with ECM regulation and cellular signaling identified as probable pathways activated upon LRP1 inhibition (Fig. 4*D*). In the chondrocyte secretome, in contrast to SMA and HEK secretomes, ECM regulation and cellular signaling pathways are likely inactivated upon LRP1 inhibition. These results suggest a different function of LRP1 in SMA tissue and HEK293 cells compared to chondrocytes but warrant further investigations since these studies used different methods to block LRP1. Studies based on RAP and LRP1 deficiency allow ligands to accumulate because the receptor is blocked or absent. Conversely, a soluble form of LRP1 binds to these ligands causing their accumulation but the ligands can be neutralized preventing them from binding to their respective receptors or other interaction partners.

### Impact of LRP1 dysfunction on the extracellular environment and cellular signaling

Loss of LRP1 function leads to perturbation of the LRP1 interactome that can have a dramatic impact on physiology. These include the accumulation of proteases in the extracellular milieu leading to increased proteolytic degradation of the matrix and modulation of cellular signaling pathways *via* the accumulation of signalling molecules. LRP1 loss also affects cellular trafficking of associated receptors. Finally, endocytic recycling and transcytosis of LRP1 ligands add further complexity of the LRP1-controlled secretome and the consequences of its dysregulation.

#### Modulation of proteolysis by accumulated LRP1 ligands

To date, several extracellular proteolytic enzymes, which cleave a broad range of substrates, have been identified as LRP1 ligands (16, 66, 86, 88) (Fig. 5*A*). Our recent studies revealed that the levels of versican, nidogen-2, and biglycan, all previously reported substrates for ADAMTS1 (169, 170, 171), were reduced in the medium of chondrocytes treated with sLRP1-II (86). Aggrecan, a major proteoglycan targeted by proteinases in cartilage, was also reduced in the chondrocyte and HEK293 secretomes (Fig. 4*C*). LRP1 is thus likely to modulate degradation of secreted proteins including ECM proteins. LRP1 mediates endocytosis of both metalloproteinases, including ADAMTSs and MMPs, and their inhibitors (88). Furthermore, LRP1 regulates α1-antitrypsin (172), an inhibitor for the potent MMP activator neutrophil elastase (173). These interactions make it difficult to predict any net outcome of LRP1-mediated endocytosis without functional studies. On the other hand, we previously showed that overexpression of sLRP1-II-N or TIMP3 reduces the proteolytic shedding of several transmembrane proteins in HEK293 cells (134). This is potentially due to the reduced activity of ADAM10 caused by accumulation of its inhibitor TIMP3. The release of certain transmembrane proteins in human chondrocytes was reduced or increased by accumulation of LRP1 ligands (98). The former is potentially due to accumulation of proteinase inhibitors as described above, whereas the latter is possibly due to increased activity of sheddases, which clearly warrants further investigations.

#### Modulation of cellular signaling by accumulated LRP1 ligands

Tissue-type plasminogen activator induces tyrosine phosphorylation of LRP1 and facilitates LRP1-mediated recruitment of β1 integrin and downstream the integrin-linked kinase signaling, leading to myofibroblast activation (159) (Fig. 5*B*). It has also been reported that RAP increases mRNA levels of proinflammatory mediators such as tumor necrosis factor α, interleukin-6, and C–C motif chemokine ligand 2 in macrophages (174). Recent studies have demonstrated that RAP or sLRP1 treatment markedly upregulates mRNA levels of LRP1 ligands MMP1 (57), MMP13 (49), and ADAMTS4 (48) as well as the non-LRP1 ligand MMP3 in human chondrocytes (86). In contrast, mRNA levels of ADAMTS5 or TIMP3 were not affected by the treatment. Cathepsin D is a lysosomal aspartic proteinase that is secreted by cells under certain physiological and pathological conditions (175). This proteinase is involved in the pathogenesis of various diseases, including breast cancer and possibly Alzheimer disease (176, 177). Cathepsin D directly binds to LRP1 β-chain (178), but not to sLRP1-II (86). However, our secretome analysis revealed that sLRP1-II treatment increased cathepsin D in the medium of chondrocytes, suggesting that extracellular availability of cathepsin D is regulated by other LRP1 ligands potentially either through indirect binding to sLRP1-II or transcriptional regulation. These observations could be the tip of the iceberg and accumulation of growth factors or other signaling molecules due to inhibition of LRP1-mediated endocytosis may alter various cellular signaling pathways, impacting not only secreted proteins but also transmembrane and intracellular proteins.

#### Modulation of cellular signaling by alteration of cellular trafficking of associated receptors

As described above, LRP1 interacts with and regulates internalisation of various cell-surface receptors and its dysfunction has significant impact on their cellular trafficking and cellular signaling (Fig. 5*C*). For example, LRP1 binds to β1 integrin *via* the cytoplasmic integrin activator kindlin-2 and mediates intracellular degradation of activated β1 integrin, regulating cell adhesion and migration on fibronectin (158). LRP1 dysfunction thus results in elevated levels of immature β1 integrin on the cell surface, disrupting its functionality. LRP1 was shown to interact with platelet-derived growth factor receptor (PDGFR)β and regulate its cell-surface levels and signaling, playing an important role in the integrity of vascular walls and cell chemotaxis (179, 180). The interaction of LRP1 and PDGFRβ also mediates PI3K activation (181), which controls actin organization and cell migration, as well as the mitogen-activated protein kinase signaling (182), a key pathway for cell proliferation and survival.

#### Endocytic clearance, recycling, and transcytosis

The majority of secreted LRP1 ligands are likely to be degraded intracellularly following internalization (28, 48, 49, 50, 86) (Fig. 5*D*). Some ligands, however, are recycled back to the extracellular milieu, facilitating their distribution and availability (29, 58, 59). Transcytosis facilitates the transcellular transport of biomolecules; internalized extracellular molecules move across the cells, and are then ejected through the opposite cell membrane by the reverse process. In brain tissue, LRP1 mediates transcytosis of amyloid-beta across the blood-brain barrier (183). It has also been shown that LRP1 mediates transcytosis of CCN2 (also known as CTGF) in chondrocytes *in vitro* (58). The fate of LRP1 ligands after internalization should have a significant impact on the LRP1-controlled secretome.

## Conclusion and perspective

The development of proteomics technologies has opened new possibilities for exploring the cell- and tissue-specific LRP1 interactome and the consequences of its disruption. However, at present, these studies are limited to only a few cell types and tissues. Moreover, little is known about the cell type and tissue specificity of the LRP1 interactome of transmembrane and intracellular adaptor proteins. The identification of an entire LRP1 interactome and LRP1-controlled secretome in specific cell/tissue/context is likely to provide novel insights into dynamic and complex biological and disease processes. The key questions in future studies are how diverse interactions of LRP1 are integrated at the cellular and tissue levels and how LRP1 dysregulation emerges. The first question can be approached by using a combination of omics methods and animal models to conditionally delete LRP1 in specific cells or tissues. One way to increase LRP1 shedding locally is to modulate the activity of proteinases known to cleave LRP1 or to overexpress soluble forms of LRP1. The second question can be approached by closely monitoring of LRP1 levels and LRP1 sheddase activities not only *in vitro* but also *ex vivo* or *in vivo* with high-resolution techniques. Additionally, knowledge on the structure of LRP1 in complexes with its various ligands will be useful in understanding how LRP1 regulates a wide variety of structurally unrelated ligands. In this regard, the recent technical advances in cryo-EM uncovered that LRP2, whose amino acid sequence is similar to LRP1, forms a homodimer that undergoes dramatic pH-dependent structural transitions that render it ligand-receptive at the cell surface and ligand-shedding in endosomes (184). Further detailed investigations of the interaction of LRP1 ligands and LRP1 may allow us to design agents able to modulate the LRP1 interactome, thereby preventing the development of diseases by altering the consequences of LRP1 dysregulation.

## Conflict of interest

The authors declare that they have no conflicts of interest with the contents of this article.
